# Rapid DNA vaccination against *Burkholderia pseudomallei* flagellin by tattoo or intranasal application

**DOI:** 10.1080/21505594.2017.1307485

**Published:** 2017-04-19

**Authors:** Jacqueline M. Lankelma, Alex Wagemakers, Emma Birnie, Bastiaan W. Haak, Jos J. A. Trentelman, Tassili A. F. Weehuizen, Jasmin Ersöz, Joris J. T. H. Roelofs, Joppe W. Hovius, W. Joost Wiersinga, Adriaan D. Bins

**Affiliations:** a Center for Experimental and Molecular Medicine, Academic Medical Center, University of Amsterdam, Amsterdam, the Netherlands; b Department of Pathology, Academic Medical Center, University of Amsterdam, Amsterdam, the Netherlands; c Department of Internal Medicine, Division of Infectious Diseases, Academic Medical Center, Amsterdam, the Netherlands

**Keywords:** *Burkholderia pseudomallei*, DNA vaccines, flagellin, melioidosis, tattoo

## Abstract

Melioidosis is a severe infectious disease with a high mortality that is endemic in South-East Asia and Northern Australia. The causative pathogen, *Burkholderia pseudomallei*, is listed as potential bioterror weapon due to its high virulence and potential for easy dissemination. Currently, there is no licensed vaccine for prevention of melioidosis. Here, we explore the use of rapid plasmid DNA vaccination against *B. pseudomallei* flagellin for protection against respiratory challenge.

We tested three flagellin DNA vaccines with different subcellular targeting designs. C57BL/6 mice were vaccinated via skin tattoo on day 0, 3 and 6 before intranasal challenge with *B. pseudomallei* on day 21. Next, the most effective construct was used as single vaccination on day 0 by tattoo or intranasal formulation. Mice were sacrificed 72 hours post-challenge to assess bacterial loads, cytokine responses, inflammation and microscopic lesions.

A construct encoding a cellular secretion signal resulted in the most effective protection against melioidosis via tattooing, with a 10-fold reduction in bacterial loads in lungs and distant organs compared to the empty vector. Strikingly, a single intranasal administration of the same vaccine resulted in >1000-fold lower bacterial loads and increased survival. Pro-inflammatory cytokine responses were significantly diminished and strong reductions in markers for distant organ damage were observed.

A rapid vaccination scheme using flagellin DNA tattoo provides significant protection against intranasal challenge with *B. pseudomallei*, markedly improved by a single administration via airway mucosa. Hence intranasal vaccination with flagellin-encoding DNA may be applicable when acute mass vaccination is indicated and warrants further testing.

## Introduction

*Burkholderia pseudomallei* is a facultative intracellular Gram negative bacterium that causes melioidosis and is endemic in Southeast Asia and Northern Australia.^1,2^ Infection usually occurs through dermal lesions via rice paddies and infected soil. During severe weather events, when the soil is disturbed and *B. pseudomallei* becomes aerosolized, inhalation is thought to cause infection. Importantly, *B. pseudomallei* was recently upgraded to a Tier 1 select agent by the National Select Agent Registry due to its high associated morbidity and mortality, intrinsic resistance to standard antimicrobial agents and lack of a vaccine. Even when treated with adequate antibiotics, mortality remains high and varies between 14 to 40% depending on geographic regions.^1,2^ Deliberate release in an aerosol form may infect large populations and poses a potential bioterrorist threat.

Despite ongoing efforts, no vaccine is currently available in humans. The requirements of a vaccination strategy for large populations that are at risk for *B. pseudomallei* bioterrorism differ from those for populations in endemic areas.^3^ While the latter requires a cost-effective vaccine primarily targeted at risk groups such as diabetics, the former should be rapidly producible and applicable in large groups of people, ideally by self-administration. In addition, the different routes of infection (i.e. aerosol distribution in case of bioterrorism) should be accounted for in the vaccination approach.^3^

Vaccination studies have focused on different methods, including live attenuated -, whole cell killed -, subunit -, recombinant - and plasmid DNA vaccines.^4,5^ Live attenuated vaccination is currently regarded as the “gold standard” in mouse models, but will have limited value in humans given the potential risk of reversion into a virulent phenotype.^4^ Vaccination with subunit and recombinant protein vaccines in Complete Freund's Adjuvant (CFA) is unattractive in humans, since the subcutaneously injected vaccine may induce painful granulomas.^6^ DNA vaccines induce both humoral and cell-mediated immune responses^7^ and have the additional benefit of being rapidly producible and having a long shelf life without the need of a cold chain.^7^

Flagellin is a suitable antigen candidate for vaccine development, as it is an important putative virulence factor of *B. pseudomallei*.^8^ It signals via Toll like receptor-5, which is upregulated in granulocytes and monocytes of patients with melioidosis.^9^ The only study so far on intramuscular DNA vaccination against *B. pseudomallei* flagellin (FliC) showed diminished bacterial loads and improved survival in mice.^10^ In this study, flagellin-encoding plasmid DNA was injected intramuscularly in BALB/c mice on day 0, 7 and 14, followed by intravenous challenge with 10^5^ CFU *B. pseudomallei* (mixture of 16 strains) eight weeks post-vaccination. In a follow-up study, using similar routes of immunization and infection, the authors reported induction of Th1-type responses as the underlying mechanism, which could be augmented by CpG oligodeoxynucleotides.^11^

Application of DNA vaccines by dermal tattoo was previously shown to improve T-cell immunity.^12^ When DNA is injected via thousands of skin perforations, an adjuvant inflammatory milieu is generated, making immune responses more robust and allowing for faster vaccination regimens.^13^ We and others have shown that rapid immunization schedules with short times between immunizations with DNA vaccines can be effective against HPV-induced tumors, viruses and bacteria, and that rapid DNA vaccination by dermal tattoo is more potent than intramuscular administration.^12-15^ Another recent development is mucosal immunization, directed at inducing tissue-resident memory T-cells.^16^ DNA vaccination via the airway mucosa would theoretically be very well-suited to prevent infection by inhalation.^7,17^ Several DNA vaccine formulations have been described in this context, one of which involving polymers such as polyethylenimine (PEI) that increase mucosal transfection by approximately thousand fold.^18-21^

In this study, we have explored rapid application of *B. pseudomallei* flagellin DNA as a possible vaccine for biodefense use, comparing dermal tattooing and intranasal administration. First, we compared multiple FliC encoding DNA vaccine designs, applied in a rapid tattoo immunization schedule. Next, using the most efficient vaccine, we tested the efficacy of intranasal administration against intranasal melioidosis.

## Results

### Designing and testing of FliC plasmid DNA vaccine candidates

**Figure 1.**
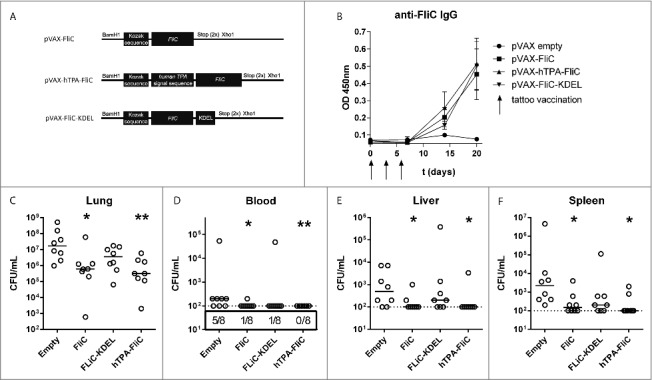
Decreased bacterial loads via rapid DNA tattooing during experimental intranasal melioidosis. (A) Schematic overview of DNA vaccine designs based on *B. pseudomallei* flagellin (FliC), cloned into the pVAX1 vector. All vaccines are codon optimized and contain a Kozak sequence to optimize ribosomal binding. hTPA-FliC contains an N-terminal cellular secretion signal, FliC-KDEL contains a C-terminal endoplasmatic reticulum retention signal. (B) FliC-specific IgG induction in plasma after rapid tattoo vaccination at t = 0, 3 and 6 days (indicated by arrows). Bars represent mean ± SEM (C-F) Three weeks after the first vaccination, mice were inoculated intranasally with 200 CFU *B. pseudomallei* and sacrificed 72 hours later. Bacterial loads in lung (C), blood (D), liver (E) and spleen homogenate (F) are depicted as scatter dot plots with a line at the median (n = 8 mice per group). Numbers in the box below (D) indicate the number of positive blood cultures/total number of mice. The vaccinated groups were compared to the empty pVAX control group using a Mann-Whitney test. *p < 0.05, **p < 0.01. For pVAX-hTPA-FliC vs. empty pVAX blood CFU a Chi-square test was performed (p = 0.007).

In order to select a DNA vaccine that rapidly protects against intranasal *B. pseudomallei* infection, we designed three constructs with FliC sequences in plasmid vector pVAX (“pVAX-FliC”). All vaccines were codon-optimized for mouse tRNA and contained a Kozak sequence to optimize translation efficiency and ribosomal binding (Fig. 1A). “pVAX-hTPA-FliC” contained an N-terminal signal peptide from human tissue plasminogen activator (hTPA), thus enabling protein secretion and augmenting MHC-II presentation by antigen presenting cells. “pVAX-FliC-KDEL” had a four-amino acid C-terminal KDEL sequence leading to FliC accumulation in the endoplasmatic reticulum (ER) of transfected cells, which via ER stress and improved MHC-I presentation may increase T-cell priming effectiveness of the vaccine. Mice were immunized by tattoo vaccination with 20 µg of plasmid DNA on day 0, 3 and 6, with a control group receiving an equal amount of empty pVAX vector via tattoo. After 20 days, all vaccine designs had induced significantly elevated anti-FliC IgG levels in plasma compared to the empty pVAX control (Fig. 1B; pVAX-FliC: p = 0.020; pVAX-hTPA-FliC: p = 0.006; pVAX-FliC-KDEL: p = 0.007). No significant differences in antibody levels were observed between FliC vaccinated groups.

On day 21 post-vaccination, mice were challenged intranasally with 200 colony forming units (CFU) of *B. pseudomallei* 1026b followed by sacrifice 72 hours after infection, when all mice usually have symptoms of systemic infection. Bacterial loads were determined in lung, blood, liver and spleen (Fig. 1C-F). Significantly reduced bacterial loads were found in both lung, blood and distant organs in mice vaccinated with pVAX-FliC and pVAX-hTPA-FliC, with the lowest bacterial concentrations in the lung observed in pVAX-hTPA-FliC vaccinated mice. In addition, all pVAX-hTPA-FliC vaccinated mice were blood-culture negative. Immunization with pVAX-FliC-KDEL was less effective than pVAX-FliC, with only a trend toward reduced bacterial loads compared to the empty pVAX control.

**Figure 2.**
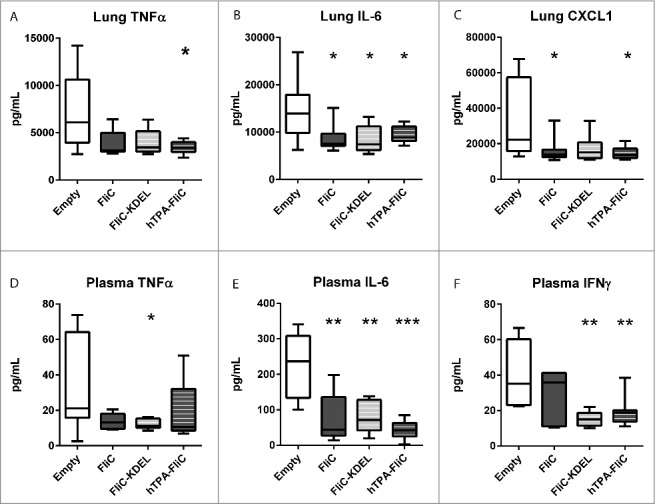
Decreased proinflammatory pulmonary and systemic cytokine levels in rapid DNA tattoo-vaccinated mice during experimental intranasal melioidosis. Mice were given rapid tattoo vaccination at t = 0, 3 and 6 days followed by intranasal bacterial challenge (200 CFU *B. pseudomallei)* on day 21. Mice were sacrificed 72 hours after intranasal *B. pseudomallei* challenge and heparinized blood plasma and lung homogenates were obtained. TNFα (A), IL-6 (B) and CXCL1 (C) were measured in lung homogenate; TNFα (D), IL-6 (E) and IFNγ (F) were measured in plasma. Values are in pg/mL; data are presented as box- and whisker plots showing the smallest observation, lower quartile, median, upper quartile and largest observation. N = 8 mice per group. Vaccinated groups were compared to the control group (empty pVAX) using a Mann Whitney test. *p < 0.05, **p < 0.01, ***p < 0.001.

Since cytokines and chemokines are important regulators of the host response during melioidosis,^8,22^ we next measured pulmonary and systemic levels of tumor necrosis factor-alpha (TNFα), interleukin (IL)-6, interferon (IFN)γ and chemokine ligand-1 (CXCL1, Fig. 2). For lung cytokine production, pVAX-hTPA-FliC was the only vaccine that significantly reduced both TNFα, IL-6 and CXCL1 (Fig. 2). Plasma cytokine concentrations were significantly reduced as well, in particular IFNγ. In contrast with the observed bacterial loads, pVAX-FliC-KDEL vaccination resulted in reduced plasma levels of TNFα, IL-6 and IFNγ, whereas pVAX-FliC only significantly decreased IL-6 levels. Intranasal inoculation with *B. pseudomallei* is associated with profound lung and distant organ pathology.^9,23,24^ A trend toward lower lung- and liver histopathologic scores was observed for all FliC vaccines, as well as a lower influx of neutrophils to the lungs as reflected by Ly6G staining, but this was not statistically significant (Fig. S1; see Supplementary Methods for histopathology scoring criteria). Combining these results, the cellular secretion signal of pVAX-hTPA-FliC appeared to improve the effectivity of the DNA vaccine. We therefore selected pVAX-hTPA-FliC as the vaccine design with the most potential for further testing.

### Single intranasal administration of pVAX-hTPA-FliC strongly reduces bacterial loads

**Figure 3.**
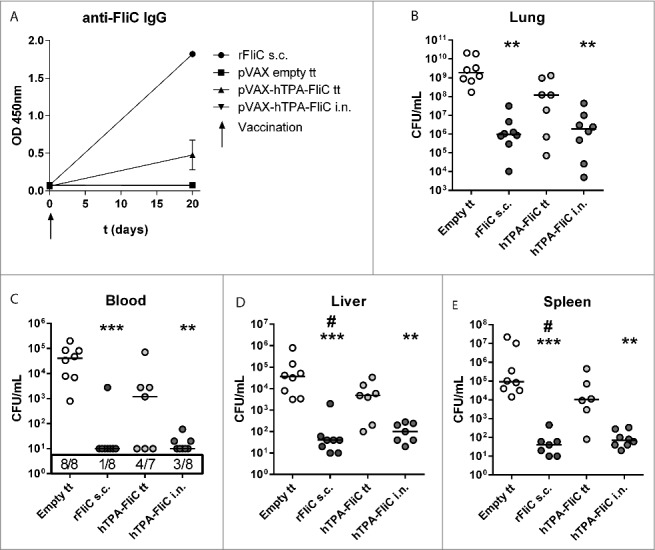
A single intranasal vaccination with pVAX-hTPA-FliC is more effective in lowering bacterial loads than single tattoo administration during experimental intranasal melioidosis. A single dose of pVAX-hTPA-FliC was administered on day 0 either via tattoo or intranasally and compared with recombinant FliC + CFA s.c. as a positive control or empty pVAX tattoo as a negative control. All mice were inoculated intranasally with 300 CFU *B. pseudomallei* on day 21. (A) FliC-specific IgG in plasma at day 0 and 20 after vaccination. Bars represent mean ± SEM (B-E) Bacterial loads in blood and organ homogenates 72 hours after infection, depicted as scatter dot plots with a line at the median. Groups were compared using a Kruskal Wallis test followed by Dunns multiple comparisons test; *p < 0.05, **p < 0.01, ***p < 0.001 versus empty pVAX; # p<0.05 vs. pVAX-hTPA-FliC tattoo. N = 7 or 8 mice per group. s.c., subcutaneous; i.n., intranasal; tt, tattoo.

Next, we were interested in the effects of a single vaccine delivery on preventing imminent intranasal *B. pseudomallei* infection. Therefore, we compared a single DNA vaccination on day 0, either via tattoo or intranasally, with recombinant FliC vaccination plus Complete Freund's Adjuvant (CFA, administered subcutaneously) as a positive control or empty pVAX tattoo as a negative control (Fig. 3). After a single pVAX-hTPA-FliC tattoo, the FliC-specific total IgG antibody response on day 20 was not as robust as after triple vaccination (Fig. 3A). No evident skewing toward IgG1 or IgG2a was detected. Interestingly, intranasal DNA vaccination did not induce a FliC- specific IgG response at all, whereas recombinant FliC with CFA induced a strong humoral response. Serum IgA was below detection in all experimental groups (data not shown).

On day 21, mice were infected with 300 CFU *B. pseudomallei* and sacrificed 72 hours later as in the previous experiment. A single tattoo vaccination with pVAX-hTPA-FliC did not lower bacterial loads, compared to the empty pVAX tattoo control group (Fig. 3B-E). However, a single intranasal application of the same DNA vaccine very effectively diminished bacterial growth and dissemination to distant organs, i.e., more than 1000-fold compared to the empty pVAX tattoo control group. This equaled the efficacy of recombinant FliC with CFA. In a separate set of experiments we compared single intranasal vaccination with pVAX-hTPA-FliC with empty pVAX intranasally and a control group without any vaccine (Fig. S2). This experiment confirmed the effectiveness of a single intranasal vaccination with pVAX-hTPA-FliC in lowering bacterial burdens. The intranasal empty pVAX vaccine formulated in PEI did not alter bacterial loads in blood or organs compared to the non-vaccinated group, confirming the antigen dependency of the pVAX-hTPA-FliC induced protection.

### Single intranasal vaccination with pVAX-hTPA-FliC reduces lung inflammation and damage

**Figure 4.**
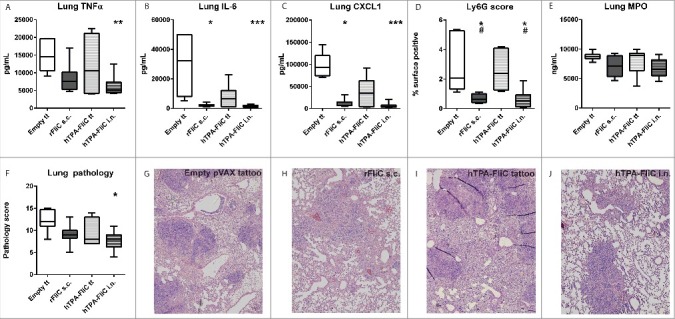
Decreased pulmonary cytokine levels and lung pathologic scores after single intranasal vaccination with pVAX-hTPA-FliC. All mice were given a single vaccination on day 0 (tattoo, subcutaneously or intranasally) and inoculated intranasally with 300 CFU *B. pseudomallei* on day 21. Lungs were harvested 72 hours after intranasal challenge with *B. pseudomallei*. TNFα (A), IL-6 (B) and CXCL1 (C) in lung homogenate; values are in pg/mL. Paraffin-embedded lung tissue sections were stained for Ly6G, a marker for neutrophil infiltration, and the percentage of the total lung surface positive for Ly6G was calculated digitally (D). As a representation for neutrophil degranulation, myeloperoxidase (MPO) was measured in lung homogenates (E). Lung tissue sections were stained with haematoxylin/eosin and scored on different parameters for pathology by a blinded pathologist (F). Representative images (with the median score) of the empty pVAX tattoo (G), rFliC + CFA (H), pVAX-hTPA-FliC tattoo (I) and pVAX-hTPA-FliC i.n. (J) groups (4x magnification). Data are presented as box- and whisker plots showing the smallest observation, lower quartile, median, upper quartile and largest observation. Groups were compared using a Kruskal Wallis test followed by Dunns multiple comparisons test; *p < 0.05, **p < 0.01, ***p < 0.001 vs. empty pVAX; # p <0.05 vs. pVAX-hTPA-FliC tattoo. N = 7 or 8 mice per group. s.c., subcutaneous; i.n., intranasal; tt, tattoo.

To further investigate the effect of vaccination on lung inflammation, we first assessed lung cytokine levels (Fig. 4A-C). Pulmonary IL-6 and CXCL1 were strongly reduced by recombinant FliC with CFA, as well as by intranasal pVAX-hTPA-FliC (p<0.001 compared to tattoo with empty pVAX vector; p = 0.06 for IL-6 and p = 0.08 for CXCL1compared to recombinant FliC with CFA). Intranasal pVAX-hTPA-FliC in addition resulted in significantly lower levels of TNFα (p<0.01 compared to empty pVAX vector, Fig. 4A). A trend toward lower IL-6 and CXCL1 was observed for single tattoo vaccination with pVAX-hTPA-FliC, but this did not reach statistical significance. We also measured neutrophil influx by staining lung sections for Ly6G, an important marker of neutrophils. As expected, the percentage of surface positive for Ly6G was significantly reduced by both intranasal pVAX-hTPA-FliC and recombinant FliC with CFA (Fig. 4D). Neutrophil degranulation, reflected by myeloperoxidase levels, was not different between the experimental groups (Fig. 4E).

Lastly, HE stained sections were scored by a blinded pathologist for several parameters of inflammation, which were combined into a lung histopathologic score (Fig. 4F; see Supplementary Methods for histopathology scoring criteria). All mice showed microscopic lesions in the lungs characterized by necrosis, interstitial inflammation, bronchitis, endothelialitis and edema 72 hours after inoculation with *B. pseudomallei*. Importantly, intranasal vaccination with pVAX-hTPA-FliC significantly reduced melioidosis- induced lung histopathology scores when compared to controls. Representative lung sections of each group are shown in Fig. 4G-J.

### Single intranasal vaccination with pVAX-hTPA-FliC reduces systemic inflammation and organ damage

**Figure 5.**
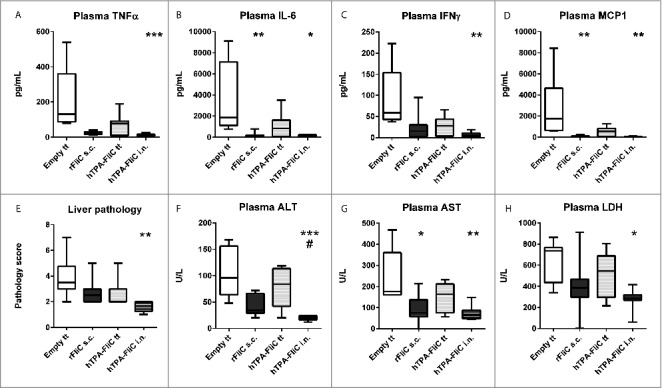
Diminished plasma cytokine levels and distant organ injury after single intranasal vaccination with pVAX-hTPA-FliC. All mice were given a single vaccination on day 0 (tattoo, subcutaneously or intranasally) and inoculated intranasally with 300 CFU *B. pseudomallei* on day 21. Plasma TNFα (A), IL-6 (B), IFNγ (C) and MCP-1 (D); values are in pg/mL. Liver tissue sections were stained with haematoxylin/eosin and scored on different parameters by a blinded pathologist, combined in a pathology score (E). ALT (F), AST (G) and LDH (H) were measured in plasma as markers for liver- and general cellular damage. Data are presented as box- and whisker plots showing the smallest observation, lower quartile, median, upper quartile and largest observation. N = 6–8 samples per group. Groups were compared using a Kruskal Wallis test followed by Dunns multiple comparisons test; *p < 0.05, **p < 0.01, ***p < 0.001 vs. empty pVAX; # p < 0.05 vs. rFliC + CFA s.c. s.c., subcutaneous; i.n., intranasal; tt, tattoo.

Systemic cytokine production followed a similar pattern as in the lungs (Fig. 5 A-D). TNFα, IL-6, IFNγ and MCP1 in plasma were all significantly lower in the intranasally pVAX-hTPA-FliC vaccinated group compared to the empty pVAX vector tattoo group, whereas for recombinant FliC in CFA this was only true for IL-6 and MCP1. A liver histopathologic score was generated in a similar way as for the lungs (Fig. 5E; see Supplementary Methods for histopathology scoring criteria). Intranasal pVAX-hTPA-FliC protected against *B. pseudomallei* induced liver pathology as reflected by strongly reduced liver histopathologic scores (Fig. 5E). In line with this finding, plasma levels of markers for hepatocellular damage alanine aminotransferase (ALT) and aspartate aminotransferase (AST) remained unaffected upon infection, in sharp contrast to the controls (Fig. 5F-G). Finally, lactate dehydrogenase (LDH) was measured in plasma as a marker for general cellular damage (Fig. 5H). Notably, this was only decreased in the intranasal pVAX-hTPA-FliC group.

### Single intranasal vaccination with pVAX-hTPA-FliC increases survival

**Figure 6.**
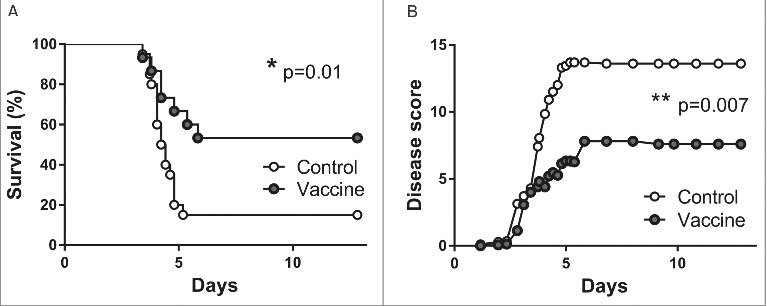
Increased survival after single intranasal vaccination with pVAX-hTPA-FliC. Survival (A) and clinical observation score (B) of control (open dots, n = 20) and intranasally vaccinated mice (gray dots, n = 15). All mice in the vaccinated group were given a single intranasal vaccination on day 0 and inoculated intranasally with 500 CFU *B. pseudomallei* on day 21. Controls did not receive any vaccine. Survival was monitored for 14 days. Data are presented as Kaplan-Meier survival curves. *p < 0.05, **p < 0.01.

To investigate whether the observed beneficial effects of intranasal vaccination would also affect outcome after intranasal infection with *B. pseudomallei*, we performed a survival experiment of 14 days. Twenty mice per group were vaccinated intranasally once with pVAX-hTPA-FliC and compared to twenty control mice that did not receive any vaccine. Unfortunately, the intranasally administered DNA vaccine led to undesired weight loss in some of the mice during this experiment, due to which four mice died and one was euthanized because of reaching a humane end point (>20% weight loss; all five were lost before the start of the survival experiment and are therefore not included in the statistics). At the moment of bacterial challenge, vaccinated- and control mice had similar weights. Intranasally vaccinated mice had significantly increased survival (53% in the vaccinated group vs. 15% in the unvaccinated control group, Fig. 6A). In addition, a clinical observation score reflects the severity of disease over time in both groups. In accordance with the survival curves, disease severity is significantly lower in the vaccinated group (Fig. 6B).

### Discussion

To our knowledge, this study is the first to investigate rapid vaccination against *B. pseudomallei* flagellin using plasmid DNA administered via tattoo or intranasally. A pVAX vector construct encoding secreted flagellin proved most effective at inducing a balanced immune response against *B. pseudomallei*, reducing bacterial burdens and improving survival without resulting in severe cytokine-induced damage. Although IFNγ is believed to play a protective role in the host defense against melioidosis,^1,25,26^ levels measured in both the systemic and pulmonary compartment during experimental melioidosis are also a reflection of disease severity; lower levels are observed in subjects with a relatively mild disease.^23,27^ As a result, the observed lower levels of IFNγ in vaccinated mice after infection with *B. pseudomallei* are most probably a reflection of a decreased inflammatory state. A single intranasal administration of the aforementioned vaccine was equally effective as subcutaneous injection of recombinant protein in CFA, strongly diminishing bacterial loads and organ damage. Intranasal DNA vaccination was the only vaccine approach that significantly diminished lung and liver pathology after a single dose. Survival of intranasally vaccinated mice was significantly increased compared to unvaccinated controls.

Single dermal DNA vaccination induced inferior IgG levels compared to the rapid immunization schedule of three immunizations within six days. Interestingly, single intranasal DNA vaccination did not induce an IgG response at all, while being more effective in lowering histopathologic scores and cytokine levels compared to recombinant protein vaccination, which induced a strong IgG response. Because intranasal DNA vaccination against *B. pseudomallei* has not been previously described, further studies are required to provide better insight into the immunological characteristics needed for such vaccines to optimally protect against melioidosis.

In the context of bioterrorism by deliberate release of aerosolized *B. pseudomallei*, a rapidly working vaccine aimed at preventing a *B. pseudomallei* pneumonia that can easily be administered can be of paramount importance. It has been described previously in mouse models of melioidosis that administration of both vaccine and bacteria via the same route induces stronger protection.^4^ Intraperitoneally administered vaccine candidates had less protective efficacy after inhalation of *B. pseudomallei* than after intraperitoneal challenge; likewise, intranasal vaccination was more effective than intraperitoneal vaccination when bacteria were administered via the nose.^28-30^ Our results support the notion that vaccination via the airways is a suitable vaccination route to protect against aerosolized *B. pseudomallei* infection, as was suggested by a number of studies.^28,31,32^ Overall, many vaccine candidates are still either considered unsafe for use in humans (live attenuated) or only induce humoral responses (purified or recombinant antigens, heat-killed whole cell vaccines) that may be insufficient to combat intracellular *B. pseudomallei* infection. DNA vaccines were shown to be effective against intracellular bacteria as well, allegedly through inducing a protective cellular immune response.^17,33,34^ The results of our study suggest that a strong cellular immune response was elicited, as we observed a strong reduction in bacterial loads through a single intranasal vaccination, with almost no detectable antibody response after the intranasal pVAX-hTPA-FliC vaccination. One important drawback of intranasal DNA vaccines is the possibility of vaccination related morbidity, manifesting as weight loss after vaccination, similar to weight loss observed after intranasal influenza infection.^14^ In young mice with a low body weight this can be life threatening. We experienced this in one of the four experiments presented in this paper (the survival experiment). Importantly, all mice - vaccinated and control mice - had similar weights at the time of bacterial challenge.

We did not include the impact of this vaccine on cell-mediated immunity in our study. Also, we did not test our vaccine in other mouse strains or in a diabetic mouse model, nor with a second strain of *B. pseudomallei*. Neither did we compare our vaccine candidate with the current “gold standard” (i.e., a live attenuated vaccine). These experiments would abide the consensus criteria for the development of melioidosis vaccines that have been published recently.^3^ However, these criteria are of special relevance in the development of a vaccine for the general population in endemic areas, as illustrated by the requirement that bacterial challenge should take place no sooner than four weeks after vaccination. Clearly, earlier challenge is specifically suitable to test the effectiveness of vaccines in an acute bioterrorism setting.

Future research should focus on understanding the underlying mechanisms of the immunity induced by intranasal DNA vaccination. In addition, it will be of interest to see whether the tattoo-administered vaccine has superior effectivity when bacteria are inoculated intradermally. The intradermal route might be more applicable to at risk populations in endemic areas, where the majority of infections are thought to occur via the cutaneous route.^1,2^ Using different vaccination regimens and routes of administration, a cost effective and safe DNA vaccine could be extremely valuable for both people at risk in endemic areas and for preparedness planning for potential deliberate release of *B. pseudomallei*.

## Material and methods

Plasmid DNA sequences, experimental infection, sample harvesting, determination of bacterial loads and assays are described in more detail in the online supplement.

### Mice

Seven-week old female specific pathogen-free C57BL/6 mice were purchased from Charles River. After one week of acclimatization, vaccination was performed at eight weeks of age. Infection was induced at eleven weeks of age. Animals were housed in IVC cages in rooms with a controlled temperature and light cyclus and received standard rodent chow and water ad libitum. The Institutional Animal Care and Use Committee of the Academic Medical Center approved all experiments.

### DNA vaccines

Three DNA vaccine inserts were designed based on the FliC gene of *B. pseudomallei* 1026b (Genbank accession U73848.1) and synthetized (Biobasic inc.). The FliC sequence was codon-optimized to murine codon usage, using JCAT web-based software.^35^ A Kozak sequence was added to all vaccines, which consists of a GCCACC before the start codon and an additional GAC triplet immediately following the start codon, leading to increased ribosomal binding. Also, a double stop codon was added. The inserts, flanked by a BamH1 and Xho1 restriction site, were cloned into pVAX1 and amplified using the Nucleobond Xtra EF kit (Macherey-Nagel) to form construct “pVAX-FliC.” Construct “pVAX-FliC-KDEL” was similarly generated with an additional 5′end AAGGACGAGCTG sequence (amino acid code: KDEL) immediately before the stop codon, thus adding an endoplasmatic reticulum retention signal. For construct “pVAX-hTPA-FliC” the start codon was immediately followed by the codon-optimized hTPA signal sequence (GenBank accession AAA61213.1), thus targeting the gene product to secretory vesicles. Full insert sequences are described in the Supplemental Material.

### Immunization

In the first experiment, vaccination was performed at day 0, 3 and 6. Eight mice per group were anesthetized using 2–4% isoflurane and their abdomens were shaved followed by additional hair removal using cream (VEET, Reckitt Benckiser). 20 µg of DNA vaccine or a control vaccine (empty pVAX) in 10 μL sterile water was administered to the naked skin and vaccination was performed for 45 seconds at 100 Hz using a tattoo machine as described previously.^13^ In the following experiments, 20 μg pVAX-hTPA-FliC or empty pVAX was administered at day 0 either via tattoo or intranasally. To optimize stability and transfection efficiency in the intranasally vaccinated group, the pVAX-hTPA-FliC was administered in 50 uL sterile, endotoxin free water with 6,5 vol/vol% polyethylenimine (PEI, PolyPlus Transfection Inc.) and 5% glucose. In the positive control group, an emulsion of 50 μg recombinant flagellin of *B. pseudomallei* 1026b (kindly provided by professor Donald E. Woods, University of Calgary, Alberta, Canada) with 50 μL Complete Freund's Adjuvant (CFA) was injected subcutaneously at two different sites at the back of the mouse.

### Induction of melioidosis and sample harvesting

Plasma was collected by tail vein bleeds at day 0, 7, 14 and 20. On day 21, experimental melioidosis was induced by intranasal inoculation with 200–500 colony forming units (CFU; approximately LD50) of *B. pseudomallei* strain 1026b in 50 µL sterile saline as described.^9,23,24^ At 72 hours post-infection, mice were euthanized (ketamine 75mg/kg and medetomedine 1,0 mg/kg intraperitoneally) and sacrificed by bleeding from the heart, after which organs were harvested.^9,23,24^ Tissues were homogenized, serially diluted and plated to assess bacterial loads (for full methods see Supplemental Material).

### Antibody responses

Induction of FliC-specific IgG was measured by coating 1 µg/mL rFliC on high-binding ELISA plates (Greiner Bio-one) overnight at 4°C, followed by blocking with 1% bovine serum albumin (BSA) in phosphate buffered saline (PBS) for 2 hours, washing with PBS-0.05%Tween, and incubation with 1:100 diluted mouse sera in 1% BSA/PBS for 1 hour. Next, plates were washed and incubated with 1:2500 anti-mouse IgG-HRP (Cell signaling) in 1% BSA-PBS for 30 minutes. After washing, plates were developed using 3,3′,5,5′-Tetramethylbenzidine substrate and read at 450 nm (Biotek).

### Survival and clinical observation score

Mice were observed for 14 days after intranasal inoculation to study survival. Clinical signs were scored as previously described:^36^ solitude (0 absent, 1 present), posture (0 normal, 1 sphere), fur (0 normal, 1 pilo-erection), eyes (0 open, 1 closed, 2 dirty), alertness (0 normal, 1 slow, 2 apathic, 3 non-responsive), pace (0 normal, 1 shaky, 2 collapse), respiration (0 normal, 1 heavy, 2 slow, 3 intermittent) and time to ascent when laid down (0 normal, 1 <5 seconds, 2 >5 seconds, 3 unresponsive); resulting in a maximum score of 16, which was also given to deceased mice. Four humane end points were enforced: if the animal was non-responsive; if time to ascent was >5 seconds, or if the animal collapsed.

### Statistical analysis

Differences between groups were analyzed by one-way ANOVA followed by Dunns multiple comparisons test or Mann Whitney test as indicated. For survival analysis, Kaplan-Meier analysis followed by log-rank test was performed. Clinical disease scores were analyzed by matched two-way ANOVA (all using GraphPad Prism 5, GraphPad Software). Values of p < 0.05 were considered statistically significant.

## Supplementary Material

KVIR_S_1307485.doc

## Abbreviations

ALT: alanine aminotransferase
AST: aspartate aminotransferase
BSA: bovine serum albumin
CFA: Complete Freund's Adjuvant
CFU: colony forming units
FliC: flagellin
HE: haematoxylin/eosin
IFN: interferon
IL: interleukin
LDH: lactate dehydrogenase
PBS: phosphate-buffered saline
PEI: polyethylenimine
TNF: tumor necrosis factor
TPA: tissue plasminogen activator
