# Supplementary material for: Rapid DNA vaccination against Burkholderia pseudomallei flagellin by tattoo or intranasal application
Source: Virulence. 2017 Apr 19;8(8):1683–94. doi: 10.1080/21505594.2017.1307485 (PMC5810493; doi:10.1080/21505594.2017.1307485)
Supplement: KVIR_S_1307485.doc [file kvir-08-08-1307485-s001.doc]

**Supplemental Material**

**Rapid DNA vaccination against *Burkholderia pseudomallei* flagellin by tattoo or intranasal application**

Jacqueline M. Lankelma^*^, Alex Wagemakers^*^, Emma Birnie, Bastiaan W. Haak, Jos J. A. Trentelman, Tassili A. F. Weehuizen, Jasmin Ersöz, Joris J. T. H. Roelofs, Joppe W. Hovius^#^, W. Joost Wiersinga ^#^, Adriaan D. Bins^#^

**SUPPLEMENTARY METHODS**

**Insert sequences**

**pVAX-FliC**

GGATCCGCCACCATGGACCTGGGCATCAACAGCAACATCAACAGCCTGGTGGCCCAGCAGAACCTGAACGGCAGCCAGGGCGCCCTGAGCCAGGCCATCACCAGGCTGAGCAGCGGCAAGAGGATCAACAGCGCCGCCGACGACGCCGCCGGCCTGGCCATCGCCACCAGGATGCAGACCCAGATCAACGGCCTGAACCAGGGCGTGAGCAACGCCAACGACGGCGTGAGCATCCTGCAGACCGCCAGCAGCGGCCTGACCAGCCTGACCAACAGCCTGCAGAGGATCAGGCAGCTGGCCGTGCAGGCCAGCAACGGCCCCCTGAGCGCCAGCGACGCCAGCGCCCTGCAGCAGGAGGTGGCCCAGCAGATCAGCGAGGTGAACAGGATCGCCAGCCAGACCAACTACAACGGCAAGAACATCCTGGACGGCAGCGCCGGCACCCTGAGCTTCCAGGTGGGCGCCAACGTGGGCCAGACCGTGAGCGTGGACCTGACCCAGAGCATGAGCGCCGCCAAGATCGGCGGCGGCATGGTGCAGACCGGCCAGACCCTGGGCACCATCAAGGTGGCCATCGACAGCAGCGGCGCCGCCTGGAGCAGCGGCAGCACCGGC

CAGGAGACCACCCAGATCAACGTGGTGAGCGACGGCAAGGGCGGCTTCACCTTCACCGACCAGAACAACCAGGCCCTGAGCAGCACCGCCGTGACCGCCGTGTTCGGCAGCAGCACCGCCGGCACCGGCACCGCCGCCAGCCCCAGCTTCCAGACCCTGGCCCTGAGCACCAGCGCCACCAGCGCCCTGAGCGCCACCGACCAGGCCAACGCCACCGCGATGGTGGCCCAGATCAACGCCGTGAACAAGCCCCAGACCGTGAGCAACCTGGACATCAGCACCCAGACCGGCGCCTACCAGGCGATGGTGAGCATCGACAACGCCCTGGCCACCGTGAACAACCTGCAGGCCACCCTGGGCGCCGCCCAGAACAGGTTCACCGCCATCGCCACCACCCAGCAGGCCGGCAGCAACAACCTGGCCCAGGCCCAGAGCCAGATCCAGAGCGCCGACTTCGCCCAGGAGACCGCCAACCTGAGCAGGGCCCAGGTGCTGCAGCAGGCCGGCATCAGCGTGCTGGCCCAGGCCAACAGCCTGCCCCAGCAGGTGCTGAAGCTGCTGCAGTAATAACTCGAG

**pVAX-hTPA-FliC**

GGATCCGCCACCATGGACGCCATGAAGAGGGGCCTGTGCTGCGTGCTGCTGCTGTGCGGCGCCGTGTTCGTGAGCCCCCTGGGCATCAACAGCAACATCAACAGCCTGGTGGCCCAGCAGAACCTGAACGGCAGCCAGGGCGCCCTGAGCCAGGCCATCACCAGGCTGAGCAGCGGCAAGAGGATCAACAGCGCCGCCGACGACGCCGCCGGCCTGGCCATCGCCACCAGGATGCAGACCCAGATCAACGGCCTGAACCAGGGCGTGAGCAACGCCAACGACGGCGTGAGCATCCTGCAGACCGCCAGCAGCGGCCTGACCAGCCTGACCAACAGCCTGCAGAGGATCAGGCAGCTGGCCGTGCAGGCCAGCAACGGCCCCCTGAGCGCCAGCGACGCCAGCGCCCTGCAGCAGGAGGTGGCCCAGCAGATCAGCGAGGTGAACAGGATCGCCAGCCAGACCAACTACAACGGCAAGAACATCCTGGACGGCAGCGCCGGCACCCTGAGCTTCCAGGTGGGCGCCAACGTGGGCCAGACCGTGAGCGTGGACCTGACCCAGAGCATGAGCGCCGCCAAGATCGGCGGCGGCATGGTGCAGACCGGCCAGACCCTGGGCACCATCAAGGTGGCCATCGACAGCAGCGGCGCCGCCTGGAGCAGCGGCAGCACCGGCCAGGAGACCACCCAGATCAACGTGGTGAGCGACGGCAAGGGCGGCTTCACCTTCACCGACCAGAACAACCAGGCCCTGAGCAGCACCGCCGTGACCGCCGTGTTCGGCAGCAGCACCGCCGGCACCGGCACCGCCGCCAGCCCCAGCTTCCAGACCCTGGCCCTGAGCACCAGCGCCACCAGCGCCCTGAGCGCCACCGA CCAGGCCAACGCCACCGCGATGGTGGCCCAGATCAACGCCGTGAACAAGCCCCAGACCGTGAGCAACCTGGACATCAGCACCCAGACCGGCGCCTACCAGGCGATGGTGAGCATCGACAACGCCCTGGCCACCGTGAACAACCTGCAGGCCACCCTGGGCGCCGCCCAGAACAGGTTCACCGCCATCGCCACCACCCAGCAGGCCGGCAGCAACAACCTGGCCCAGGCCCAGAGCCAGATCCAGAGCGCCGACTTCGCCCAGGAGACCGCCAACCTGAGCAGGGCCCAGGTGCTGCAGCAGGCCGGCATCAGCGTGCTGGCCCAGGCCAACAGCCTGCCCCAGCAGGTGCTGAAGCTGCTGCAGTAATAACTCGAG

**pVAX-FliC-KDEL**

GGATCCGCCACCATGGACCTGGGCATCAACAGCAACATCAACAGCCTGGTGGCCCAGCAGAACCTGAACGGCAGCCAGGGCGCCCTGAGCCAGGCCATCACCAGGCTGAGCAGCGGCAAGAGGATCAACAGCGCCGCCGACGACGCCGCCGGCCTGGCCATCGCCACCAGGATGCAGACCCAGATCAACGGCCTGAACCAGGGCGTGAGCAACGCCAACGACGGCGTGAGCATCCTGCAGACCGCCAGCAGCGGCCTGACCAGCCTGACCAACAGCCTGCAGAGGATCAGGCAGCTGGCCGTGCAGGCCAGCAACGGCCCCCTGAGCGCCAGCGACGCCAGCGCCCTGCAGCAGGAGGTGGCCCAGCAGATCAGCGAGGTGAACAGGATCGCCAGCCAGACCAACTACAACGGCAAGAACATCCTGGACGGCAGCGCCGGCACCCTGAGCTTCCAGGTGGGCGCCAACGTGGGCCAGACCGTGAGCGTGGACCTGACCCAGAGCATGAGCGCCGCCAAGATCGGCGGCGGCATGGTGCAGACCGGCCAGACCCTGGGCACCATCAAGGTGGCCATCGACAGCAGCGGCGCCGCCTGGAGCAGCGGCAGCACCGGC

CAGGAGACCACCCAGATCAACGTGGTGAGCGACGGCAAGGGCGGCTTCACCTTCACCGACCAGAACAACCAGGCCCTGAGCAGCACCGCCGTGACCGCCGTGTTCGGCAGCAGCACCGCCGGCACCGGCACCGCCGCCAGCCCCAGCTTCCAGACCCTGGCCCTGAGCACCAGCGCCACCAGCGCCCTGAGCGCCACCGACCAGGCCAACGCCACCGCGATGGTGGCCCAGATCAACGCCGTGAACAAGCCCCAGACCGTGAGCAACCTGGACATCAGCACCCAGACCGGCGCCTACCAGGCGATGGTGAGCATCGACAACGCCCTGGCCACCGTGAACAACCTGCAGGCCACCCTGGGCGCCGCCCAGAACAGGTTCACCGCCATCGCCACCACCCAGCAGGCCGGCAGCAACAACCTGGCCCAGGCCCAGAGCCAGATCCAGAGCGCCGACTTCGCCCAGGAGACCGCCAACCTGAGCAGGGCCCAGGTGCTGCAGCAGGCCGGCATCAGCGTGCTGGCCCAGGCCAACAGCCTGCCCCAGCAGGTGCTGAAGCTGCTGCAGAAGGACGAGCTGTAATAACTCGAG

**Experimental infection**

*B. pseudomallei* strain 1026b, isolated in 1993 from a blood culture from a septic 29-year old female rice farmer in Sappasithiprasong hospital, was grown from frozen aliquots in 50 mL Luria Broth overnight at 37°C in a shaking 5% CO2 incubator. One mL of this culture was transferred to fresh Luria Broth and grown for about three hours to midlogarithmic phase. Bacteria were diluted in sterile saline to a concentration of 200-400 CFU/ 50 uL, as determined by plating serial dilutions on blood agar plates. Pneumonia was induced by intranasal inoculation of 50 uL bacterial suspension after inhalation anesthesia with isoflurane (2-3% in 100% oxygen).

**Sample collection and determination of bacterial loads**

Mice were euthanized by intraperitoneal injection of ketamine/ dexmedetomidine 72 hours post-infection (n=6-8 mice per group). Blood was drawn by cardiac puncture, transferred to heparin Vacutainer tubes, and immediately cooled. Broncho-alveolar lavage was performed using 1 mL phosphate buffered saline (PBS). Next, the abdomen was opened and liver, spleen and lungs were harvested. Lung, spleen and liver were homogenised in four volumes of sterile isotonic saline. For bacterial quantification, blood and organ homogenates were serially 10-fold diluted in sterile isotonic saline and 50 uL of each dilution was plated onto blood agar plates. Following 24h of incubation at 37°C, CFUs were counted and corrected for dilution. For cytokine measurements, lung homogenates were diluted 1:1 with Greenberger lysis buffer (300 mM NaCl, 30 mM Tris, 2 mM MgCl2, 2 mM CaCl2, 1% (v/v) Triton X-100, pH 7.4) with protease inhibitor mix (Complete protease inhibitor cocktail tablets, Roche) and incubated for 30 min on ice, followed by centrifugation at 680 g for 10 min. Supernatants were stored at -20°C until analysis.

**Histology**

Directly after sacrifice, lungs and livers were fixed in 4% formalin and embedded in paraffin. Sections of 4um thickness were stained with haematoxylin and eosin. All slides were coded and scored by an experienced, blinded pathologist. Lungs were graded for bronchitis, edema, interstitial inflammation, necrosis, pleuritis, endothelialitis, thrombus formation and percentage of tissue surface area involved on a scale of 0 to 4, with 0 as “absent” and 4 as “severe.” The total lung inflammation score is expressed as the sum of the scores for each parameter. Liver sections were scored on parenchymal inflammation, necrosis and thrombus formation using the scale given above with a maximum score of 12. Granulocyte staining was performed using FITC-labelled rat anti-mouse Ly-6G (Pharmingen), counterstained with methylgreen. Lung tissue sections were scanned with an Olympus Slide system and Ly-6G staining was quantified by digital image analysis (ImageJ); the amount of Ly-6G positivity is expressed as a percentage of the total surface area.

**Assays**

TNF-α, IL-6, MCP-1 and IFN-g were measured in plasma by cytometric bead array (mouse inflammation kit, BD Biosciences) in accordance with the manufacturers’ recommendations. Cytokines and chemokines in lung homogenate and BALF were determined by enzyme linked immunosorbent assays (ELISA; R&D Systems, Minneapolis, MN) according to the manufacturer’s instructions. Lactate dehydrogenase (LDH), aspartate aminotransferase (AST) and alanine aminotransferase (ALT) were measured using a c702 Roche Diagnostics machine.

**SUPPLEMENTARY FIGURES**

**
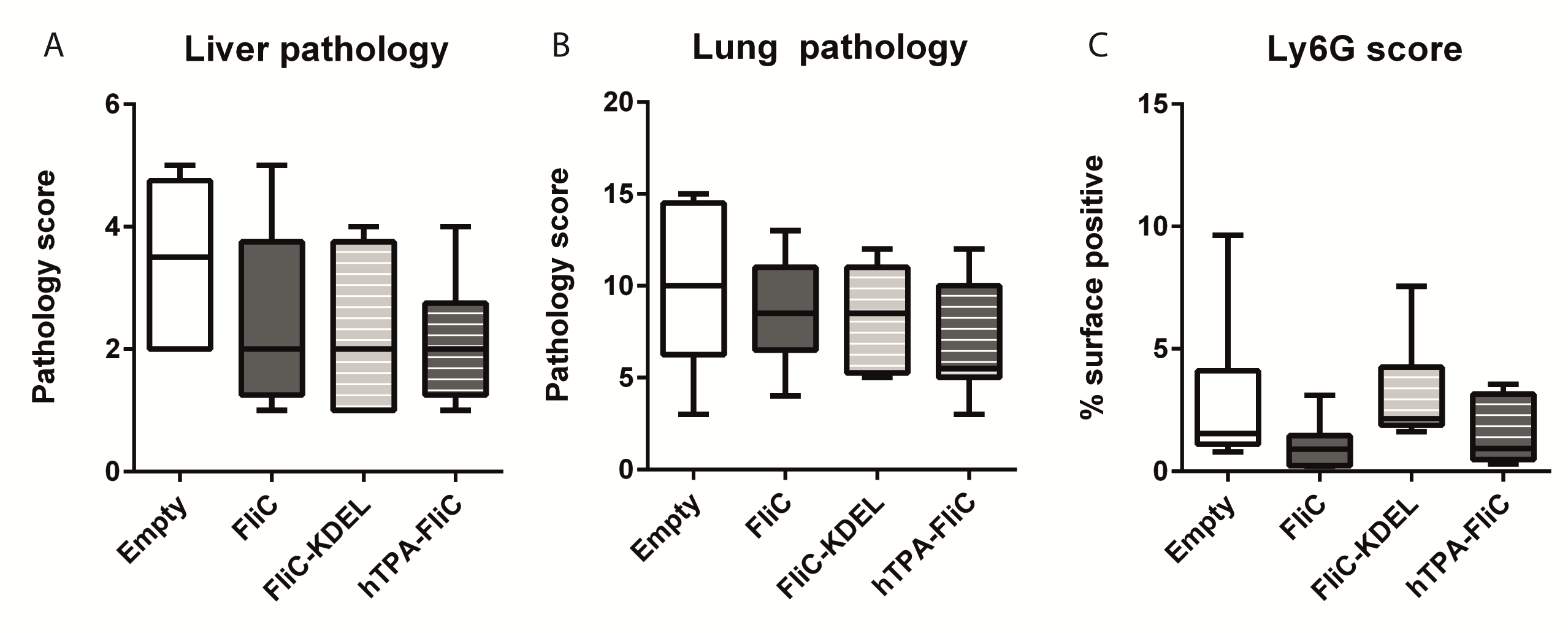
**

**Supplementary Figure 1. Organ damage in rapid DNA tattoo- vaccinated mice during experimental intranasal melioidosis.** Mice were given rapid tattoo vaccination at t=0, 3 and 6 days followed by intranasal bacterial challenge on day 21. Mice were sacrificed 72 hours after intranasal *B. pseudomallei* challenge and livers and lungs were obtained. Paraffin-embedded lung tissue sections were stained with haematoxylin/eosin and scored on different parameters by a blinded pathologist, combined in a pathology score for liver (A) and lung (B). Sections from the same lung samples were stained for Ly6G, a marker for neutrophil infiltration, and the percentage of the total lung surface positive for Ly6G was calculated digitally (C). Data are presented as box- and whisker plots showing the smallest observation, lower quartile, median, upper quartile and largest observation. N= 8 mice per group. No statistically significant differences were observed.

**
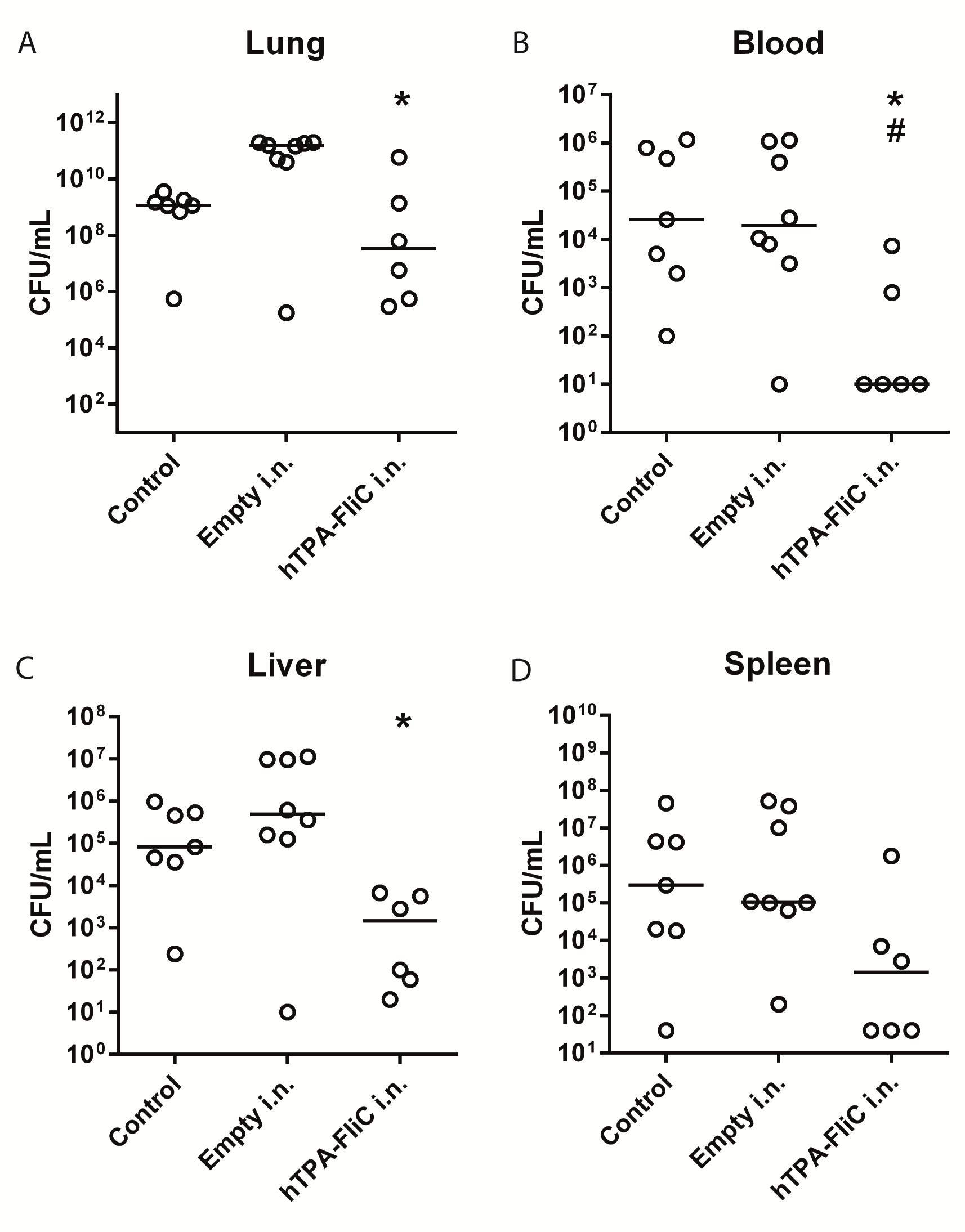
**

**Supplementary Figure 2. No effect of intranasal vaccination with empty pVAX on bacterial counts during experimental intranasal melioidosis.** A single dose of pVAX-hTPA-FliC or empty pVAX was administered intranasally on day 0, followed by intranasal inoculation with 400 CFU *B. pseudomallei* on day 21. The control group did not receive any vaccine. Mice were sacrificed 72 hours after intranasal *B. pseudomallei* challenge and lung, blood, liver and lung were obtained. (A) Lung, (B) blood, (C) liver and (D) spleen bacterial loads, 72 hours after infection, depicted as scatter dot plots with a line at the median. Groups were compared using a Kruskal Wallis test followed by Dunns multiple comparisons test; * p<0.05 versus empty pVAX i.n.; # p<0.05 versus control. N= 7 or 8 mice per group; in both the control- and pVAX-hTPA-FliC group, one mouse was found dead at t=72h.
